# Ventriculostomy-associated infections: a healthcare issue in the neurointensive care unit

**DOI:** 10.62675/2965-2774.20250250ed

**Published:** 2025-01-15

**Authors:** Viviane Cordeiro Veiga, André Kalil, Pedro Henrique Rigotti Soares, Pedro Póvoa

**Affiliations:** 1 Beneficência Portuguesa de São Paulo São Paulo SP Brazil BP - A Beneficência Portuguesa de São Paulo - São Paulo (SP), Brazil.; 2 University of Nebraska Medical Center Omaha Nebraska United States University of Nebraska Medical Center - Omaha, Nebraska, United States.; 3 Hospital Nossa Senhora da Conceição Porto Alegre RS Brazil Hospital Nossa Senhora da Conceição - Porto Alegre (RS), Brazil.; 4 Hospital São Francisco Xavier Centro Hospitalar Lisboa Ocidental Lisbon Portugal Hospital São Francisco Xavier, Centro Hospitalar Lisboa Ocidental - Lisbon, Portugal.

Healthcare-associated infections (HAIs) are a significant challenge in hospital settings, leading to increased morbidity and mortality among patients. Importantly, many instances of HAIs are preventable. Extensive observational research has revealed that the prevalence of HAIs is 5 to 10% in the United States and Europe, but it has increased to approximately 40% in regions such as Latin America, Sub-Saharan Africa, and Asia. The use of invasive medical devices, which are ubiquitous in the intensive care unit (ICU), increases the risk of these complications. This risk is also significant in the neurointensive ICU (neuro-ICU), where patients receive specialized care in challenging conditions such as coma, frequent aspirations, and the need for invasive medical devices.^1-3^

The most common HAIs in the neuro-ICU are urinary tract infections (UTIs), pneumonia, and ventriculostomy-associated infections (VAIs). Ventriculostomy-associated infections are particularly relevant in neuro-ICU patients with subarachnoid hemorrhage (SAH) because of the insertion of an external ventricular drain (EVD). The main risk factors for VAIs are diabetes, Hunt and Hess III-V, intraventricular hemorrhage, and catheter manipulation. The implementation of care bundles during insertion and manipulation may help reduce the incidence of VAIs, although this hypothesis has not been widely studied. Extensive observational studies have shown an association between VAIs and increased mortality in these patients, but further research is crucial to fully understand this association.^3-5^

The diagnosis of ventriculitis remains a challenge in our setting, with the incidence varying in the literature from 2.6% to 36%,^1,6-10^depending on the diagnostic criteria used and the considerable variability of healthcare resources, especially in patients from low- and middle-income countries (LMICs). Although the diagnostic criteria of the Centers for Disease Control and Prevention (CDC) are widely used, they are not universally adopted, and compared with other criteria,^11-13^they are associated with a greater number of diagnoses.^14^

Turon et al. performed a prospective observational study to define the incidence of VAI and assess its impact on mortality and functional outcomes (short- and long-term) in patients with SAH with aneurysmal etiology.^15^ They included all consecutive patients with SAH admitted to 2 neuro-ICUs of reference centers in Brazil that required EVD from July 2015 to December 2020. A total of 271 out of 676 patients with SAH required EVD. The presence of a VAI was evaluated daily via the CDC criteria. They reported that 47% (n = 127) of patients developed VAI, which had no impact on hospital or 12-month mortality (VAI 36% *versus* non-VAI 40% and VAI 43% *versus* non-VAI 49%, respectively) or on on functional outcomes (modified Rankin scale) at hospital discharge and after 12 months (VAI 75% *versus* non-VAI 73%, p = NS; VAI 49% *versus* non-VAI 53%, p = NS, respectively). As the authors stated in the discussion, this seems to be a higher than expected incidence of VAI; however, there is no information concerning the use of antibiotic prophylaxis during EVD insertion or the adherence to this protocol.^16,17^

Another limitation is the absence of data concerning the prescription of antibiotic therapy during the ICU stay, which could have had a marked influence on the rate of microbiologic identification (only 19%) as well as on the pathogens identified as gram-negative instead of the more expected gram-positive pathogens, according to the literature. Although there is no recommendation for that procedure, the authors assessed daily the presence of VAI criteria independent of the presence or absence of clinical suspicion that could lead to a high rate of false-positives and, consequently, to a small/negligible attributable mortality and impact on functional outcomes.

Ventriculostomy-associated infections are expected to be associated with worse outcomes, i.e., higher morbidity and mortality in patients with SAH, but this was not observed in this new study. There are four main interpretations of these results: 1) VAIs are not associated with worse outcomes. However, to reach this conclusion, the study needs to have statistical power to detect differences, be free of bias and confounding factors, and achieve an accurate diagnosis of VAI. Thus, three other interpretations exist: 2) The study was underpowered. This remains possible because of the small sample size and the small difference in outcome estimates between VAIs and non-VAIs. 3) The study results were affected by unexpected selection bias and confounding factors. Selection bias is likely because a major baseline factor, the World Federation of Neurological Surgeons (WFNS) grade, which also influences the primary objective (functional status outcome) of the study, was significantly imbalanced between groups as follows: WFNS Grade V (17% VAI *versus* 28% non-VAI); Grade IV-V – Definition of Poor Grade (43% *versus* 52%). This baseline imbalance alone biased the study functional outcome against the non-VAI group, which in turn made the VAI group look better than expected. In accordance with these findings, the non-VAI group had a greater incidence of hydrocephalus, mechanical ventilation, delayed cerebral ischemia, and hospital mortality compared to that in the VAI group. This selection bias cannot be corrected by regression analysis alone, particularly considering that the models could not have more than a few predictor variables due to the low absolute number of events. 4) The accuracy of the study VAI diagnosis is limited because only 19% of the cerebrospinal fluid cultures had a positive culture. Thus, most patients may not have had a true VAI, which could have led to the absence of outcome differences between the groups. Large multicenter prospective cohort studies with sensitive and specific microbiological tests are necessary to address these unanswered questions. We congratulate the study’s authors for their important work and the relevant addition of knowledge to a field in need of more evidence.
